# Wavelet-enhanced autoencoder based image denoising with CNN fusion

**DOI:** 10.1038/s41598-026-47179-1

**Published:** 2026-04-06

**Authors:** Iclal Cetin Tas

**Affiliations:** Department of Computer Engineering, Baskent University, Etimesgut, 06790 Ankara, Turkey

**Keywords:** Medical images, Wavelet, Autoencoder, CNN fusion, Noise reduction, Computational biology and bioinformatics, Engineering, Mathematics and computing

## Abstract

In biomedical imaging, noise is a fundamental problem negatively impacting diagnostic quality, and effective noise reduction methods are critical for preserving structural details. This study proposes a hybrid noise reduction framework integrated with a CNN-based fusion network, combining a convolutional autoencoder, a specially designed hybrid wavelet filtering approach, and multi-level adaptive wavelet transformation. The autoencoder component learns clean image matches from noisy inputs, while the wavelet-based arm ensures the preservation of high-frequency structural and textural information through multi-level decomposition. The CNN-based fusion module adaptively combines these complementary representations to produce the final enhanced image. Experimental results demonstrate that the proposed method provides consistent superiority across different noise types and datasets. In the fetal ultrasound dataset, the highest performance was achieved at all levels under Gaussian and speckle noise; for speckle noise low-level PSNR values of 48.12 dB and SSIM of 0.99 were obtained. For brain tumor MR dataset, the method was effective not only under Gaussian and speckle noise but also under Rician noise; the PSNR value was obtained as 22.90 dB at the low level, 27.11 dB at the medium level, and 22.17 dB at the high level, with corresponding SSIM values of 0.90, 0.59, and 0.47. Furthermore, in non-referenced evaluations performed on the wrist trauma clinical X-ray dataset, mean NIQE = 3.12 and SSEQ = 0.26 values were obtained. These results confirm that the proposed approach offers balanced and generalizable denoising performance without excessive smoothing or the creation of artificial artifacts on real clinical images. These findings demonstrate that combining wavelet-based detail preservation with deep learning-based reconstruction delivers robust and reliable noise reduction performance across diverse biomedical imaging modalities and noise types.

## Introduction

Medical imaging systems are an integral part of the early diagnosis and treatment of diseases. However, working with healthy images is not always possible. The resulting images are often corrupted by noise from various sources, which negatively impacts diagnostic accuracy. Noise can arise from factors such as device hardware, environmental factors, and patient movement. Therefore, developing effective denoising techniques in medical images is vital for improving image quality and ensuring an accurate and timely diagnosis. Identifying the noise factor is also a critical step. Artificial neural network (NN)-based methods have significantly transformed medical image processing in recent years, playing a decisive role, particularly in denoising, image enhancement, segmentation, and diagnostic support systems. The high-accuracy feature extraction and complex relationship learning capacity of NNs are critical for improving the diagnostic quality of medical images across modalities (MRI, CT, USG, etc.). New NN architectures implements for hardware, when considered in conjunction with memristor-based circuit solutions inspired by biological learning processes, enable AI-supported medical imaging systems to operate faster, more reliably, and more energy-efficiently. Recent studies demonstrate that high performance can be achieved in real-time autonomous applications by combining memristive NN structures with advanced cognitive mechanisms such as multi-objective decision making, reward-punishment learning, and contextual revaluation^1–3^. These advances further increase the importance of NN-based models for medical image enhancement and diagnostic accuracy, and encourage the development of new hybrid approaches in the literature. Algorithms for denoising range from iterative reconstruction techniques to the currently widely used deep learning (DL)-based approaches. Rapid advances in artificial intelligence techniques require comprehensive analysis of recently developed methods. In this context, it is important to focus not only on performance comparisons but also on critical elements such as model training, validation, testing processes, robustness, security, and evaluation methods. Such reviews highlight the multifaceted challenges encountered in developing DL-based CT denoising models and highlight the need for optimization for clinical applicability^4^. In recent years, machine learning (ML) and DL-based denoising approaches have reached maturity as a powerful alternative to traditional model-based techniques. While traditional methods require explicitly estimating the noise distribution in an image, DL-based models demonstrate higher adaptability by directly learning complex transformations between noisy input and clean output from large datasets. Autoencoder-based denoising methods are particularly noteworthy. The autoencoder architecture consists of an encoder that transforms the noisy input image into a low-dimensional latent space and a decoder that performs a reconstruction from this representation. This mechanism allows for the suppression of random noise components while preserving structural information. Successful applications of autoencoders to the reduction of Poisson noise and Rician noise have been reported in the literature. This shows that autoencoder-based models represent a promising but still developing research direction in the field of adaptive medical image denoising^5,6^. The unsupervised two-stage autoencoder-based surface defect detection model proposed by Shiferaw and Yao^7^ achieved 89.5% detection accuracy for unlabeled data. Furthermore, the model reduced processing time by 20% compared to previous methods and minimized error rates. This contributed to accelerating quality control and reducing costs in manufacturing processes.

Deep learning-based methods, especially autoencoder architectures, are becoming increasingly common in medical image processing and disease prediction. Bhute et al.^8^ developed skip-connected autoencoders to reduce speckle noise in ultrasound images. The proposed model achieved up to 18% noise reduction compared to conventional filtering methods, resulting in a significant improvement in image quality. Furthermore, the structure of the model prevented significant information loss and preserved details. Gu et al.^9^ used deep autoencoders to remove noise from experimental image data without clean target data, achieving noise reductions of up to 15% (based on PSNR and SSIM metrics). This method significantly improved data quality by reducing noise-induced signal distortion, particularly in biomedical experiments, thus setting a new standard for systems operating in noisy environments.

Byeon et al.^10^ reported that the model they developed, combining autoencoders and extensive learning, achieved success rates greater than 90% in various biomedical data types. The model was found to be suitable for use in clinical applications with its privacy-protecting features for patients and stands out as a reliable solution for early disease diagnosis. These advances demonstrate the effectiveness and potential of deep learning in medical image processing and diagnostic support systems. In this study, we propose a hybrid, multi-stage deep learning-based method for denoising medical images. The proposed approach begins with hybrid wavelet denoising, which combines the strengths of different wavelet families. It then combines these outputs with a multi-layer autoencoder to enhance image quality, and finally fuses them with a deep convolutional neural network (CNN) to achieve superior restoration performance. This multi-layer structure overcomes the limitations of single methods and effectively reduces both structural and statistical noise components. Experimental results are compared to literature using PSNR and SSIM metrics.Let’s briefly summarize the innovative aspects of the study that contribute to the literature.

- Hybrid wavelet denoising: in this study, the best denoising results from two different wavelet families were blended using a PSNR-weighted proportional method, achieving performance improvements beyond the traditional single wavelet method.
- Deep autoencoder design: an optimized autoencoder architecture with multilayer and regularized layer learning rates was developed to reconstruct the missing information in noisy images, thus preserving structural details.
- Post-enhancement with a CNN fusion network: high-quality, noise-free image reconstruction was achieved by combining hybrid wavelet and autoencoder outputs with a custom-designed CNN model used as two-channel input; this approach is a multi-stage fusion strategy rarely used in the literature.

In this study, a multilayered method for denoising medical images is proposed. The materials section describes the datasets and experimental setting in detail. The methods section discusses wavelet-based filtering, autoencoder, and the proposed deep CNN architecture for image denoising. The results and discussion section presents a comparison of the methods, performance metrics, analysis, and resulting improvements. Finally, the general findings of the study and future recommendations are summarized in the conclusion section.

## Key contributions

The main contributions of this study are summarized as follows:

- A novel hybrid denoising framework that integrates wavelet-domain thresholding, a patch-based autoencoder, and a CNN-based fusion module, enable complementary exploitation of multi-resolution frequency information and deep spatial feature learning.
- A PSNR-weighted multi–wavelet fusion strategy, where the two most informative wavelet bases are automatically selected and adaptively combined based on their individual reconstruction performance, improving detail preservation under speckle noise.
- A training paradigm based on patch-level learning, which enhances the model’s ability to restore fine anatomical structures and boundaries, particularly important in fetal ultrasound imaging.
- Demonstration that the proposed hybrid Wavelet–Autoencoder–CNN pipeline outperforms single-domain (wavelet only) and single-model (autoencoder only) approaches, supported by a systematic ablation study.
- A lightweight and computationally efficient architecture suitable for real–time or clinical deployment, avoiding complex diffusion or transformer models while retaining high denoising accuracy.
- To the best of our knowledge, the first implementation of a PSNR-weighted multi-wavelet + autoencoder + CNN fusion strategy for speckle suppression in fetal ultrasound imaging, highlighting its potential for improved visual clarity in medical diagnostics.
- The introduction of an adaptive, data-driven fusion mechanism that learns how much to rely on the wavelet branch versus the autoencoder branch at each spatial location, enabled by a 2-channel patch-level CNN fusion architecture. This learned fusion replaces fixed combination rules and provides localized, content-aware denoising.
- Comprehensive explainability and validation through ablation experiments and feature map visualization, demonstrating that the CNN fusion module effectively leverages the complementary strengths of both domains-preserving structural edges from the wavelet branch while suppressing noise patterns captured by the autoencoder-ultimately confirming that the hybrid design, not model complexity, drives the performance gains.

## Materials and methods

All operations related to image processing, optimization and performance evaluation were carried out using MATLAB version 2025a, and the studies were performed on an ASUS ZenBook 14 laptop with 16 GB RAM and an Intel Core i9 processor. In the field of medical imaging, ultrasound and MRI images, in particular, frequently encounter noise problems due to low contrast and device-induced noise. Hence, data frequently used in image enhancement and denoising studies. Thus, to increase robustness, the study utilized the Fetal Head Ultrasound Dataset^11^ on the Kaggle platform and the brain tumor MRI dataset^12^ on Figshare . The first dataset consists of fetal head ultrasound images.It contains 3064 samples in grayscale .png format. When using fetal dataset, the annotated copies in it were removed. The total number of samples was 1262. The dataset consists of T1, T2, and FLAIR sequences of different tumor types, including glioma, meningioma, and pituitary adenoma and no tumor samples. The images are grayscale.Total samples 2868. During model training, 80% of the datasets were used for training and 20% for testing. In this study, medical images with various types and levels of synthetic noise were used to evaluate the effectiveness of the proposed denoising approach. High-resolution magnetic resonance imaging (MRI) and ultrasound scans were chosen as test data. Medical imaging methods are subject to various types of noise, depending on each imaging modality, the physical principles used, and the device infrastructure. This noise reduces image quality, making visual inspection difficult and negatively impacting the performance of computer-aided diagnostic systems. Therefore, a comparative analysis of the types of noise generated by different imaging techniques and their implications for clinical practice is crucial for developing denoising methods. Table 1 briefly summarizes the image types and the resulting noise types.

**Table 1:** Summary of datasets used in the experiments.

Dataset	Type	Samples	Resolution	Noise
Brain Tumor MRI (Figshare)	MRI	3064	$512\times 512$	Gauss, Speckle
Fetal Head Ultrasound (Kaggle)	US	1262	$540\times 800$	Gauss, Speckle

DOI: 10.6084/m9.figshare.1512427.v5.

https://www.kaggle.com/datasets/ankit8467/fetal-head-ultrasound-dataset-for-image-segment.

In this study, MRI and ultrasound images were chosen for medical image processing and denoising applications. MRI images were selected due to their high soft tissue contrast and the ability to examine the brain, spinal cord, joints, and other soft tissues in detail. While thermal and system noise observed in MRI data can be statistically modeled with Gaussian noise, distortions such as motion artifacts require advanced restoration techniques. Ultrasound images are used in common clinical applications such as obstetrics, cardiology, and abdominal imaging, and speckle noise, in particular, degrades image quality when combined with electronic and acoustic noise. Speckle noise was applied to ultrasound images to provide a simulation close to real-world clinical conditions. Therefore, evaluations conducted on images obtained from different modalities and various noise types will demonstrate both the robustness and suitability of the proposed denoising methods for clinical applications.

Two different noise models frequently encountered in clinical scenarios were synthetically generated on the original images:

Gaussian noise: White Gaussian noise with zero mean and different variance values was added to represent sensor reading errors, thermal noise, and random fluctuations caused by electronic circuitry in MRI and CT images.

Speckle noise: Multiplicative speckle noise was added to mimic multiplicative noise caused by multiple reflections, particularly in ultrasound and coherent imaging techniques.

**Fig. 1:**
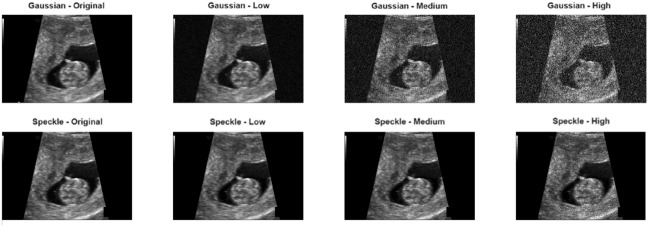
Noise types and with different intensities for fetal ultrasound dataset.

**Fig. 2:**
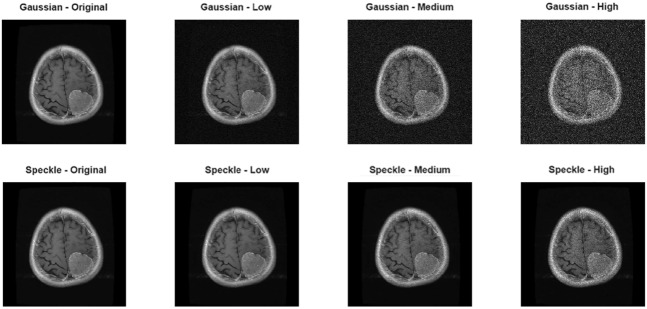
Noise types and with different intensities for brain tumor MR dataset.

Each noise type was applied at three different intensity levels to examine the performance of method under low, medium, and high distortion conditions. For Gaussian and Speckle noise variance values are 0.003, 0.03, and 0.1. To simulate realistic ultrasound distortion, three noise levels were selected to represent low, moderate, and severe speckle/Gaussian distortion conditions frequently observed in clinical fetal imaging. Previous studies report that fetal and abdominal ultrasound images typically exhibit speckle variances in a normalized range of 0.002–0.02 under standard acquisition settings due to tissue scattering and electronic noise. Therefore, a noise level of 0.003 corresponds to subtle but clinically present speckle patterns^13,14^. The midlevel of 0.03 has been widely used in simulation studies to represent moderate levels of speckle intensity that begin to blur anatomical boundaries^15,16^. The high noise level of 0.1 reflects degraded imaging conditions known to occur with low-quality probes, suboptimal fetal positions, or high attenuation scenarios^17,18^. Using these three representative noise levels provides an assessment of the clinical noise intensity of the proposed method that is controlled but reflective of real-world conditions, enabling assessment of both the preservation of fine detail and robustness under challenging acquisition conditions. Noise additions were performed using the built-in imnoise function in MATLAB R2025a. These parameters were selected from values representative of distortion levels commonly used in the literature and those encountered in clinical images. The sample images presented are both original, noise-free versions and versions with added noise (in Figs. 1 and 2).

**Fig. 3:**
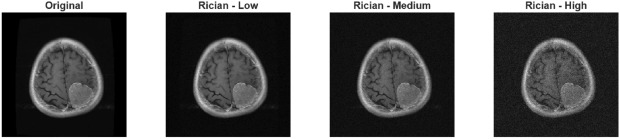
Rician noise with different intensities for brain tumor MR dataset.

Rician noise is one of the most common types of noise in MRI systems. Due to the complex-valued nature of the signal and the amplitude reconstruction process, it exhibits a characteristic noise distribution. Therefore, in addition to other experiments, a Rician noise model was used in this study to represent realistic noise conditions in MRI images. MRI images are affected by noise originating from the magnitude reconstruction of complex-valued signals, which results in a Rician-distributed noise pattern. In this study, Rician noise was generated synthetically according to Eq. 1, while its statistical properties are described by the probability density function given in Eq. 2. Figure 3shows a sample MRI image with Rician noise applied.

1 $$\begin{aligned} I_R = \sqrt{(I + n_1)^2 + n_2^2} \end{aligned}$$

*I* denotes the noise-free (ideal) MRI image, $n_1, n_2 \sim {\mathcal {N}}(0,\sigma ^2)$ are independent zero-mean Gaussian noise components added to the real and imaginary channels, respectively, $\sigma$ represents the standard deviation of the noise, $I_R$ is the resulting MRI image corrupted by Rician noise.

2 $$\begin{aligned} p(x \mid \nu , \sigma ) = \frac{x}{\sigma ^{2}} \exp \left( -\frac{x^{2} + \nu ^{2}}{2\sigma ^{2}} \right) I_{0}\left( \frac{x \nu }{\sigma ^{2}} \right) , \quad x \ge 0 \end{aligned}$$

*x* denotes the observed magnitude value of a pixel, $\nu$ represents the true (noise-free) signal amplitude, $\sigma$ is the standard deviation of the underlying Gaussian noise, $I_0(\cdot )$ denotes the modified Bessel function of the first kind and zero order.

**Fig. 4:**
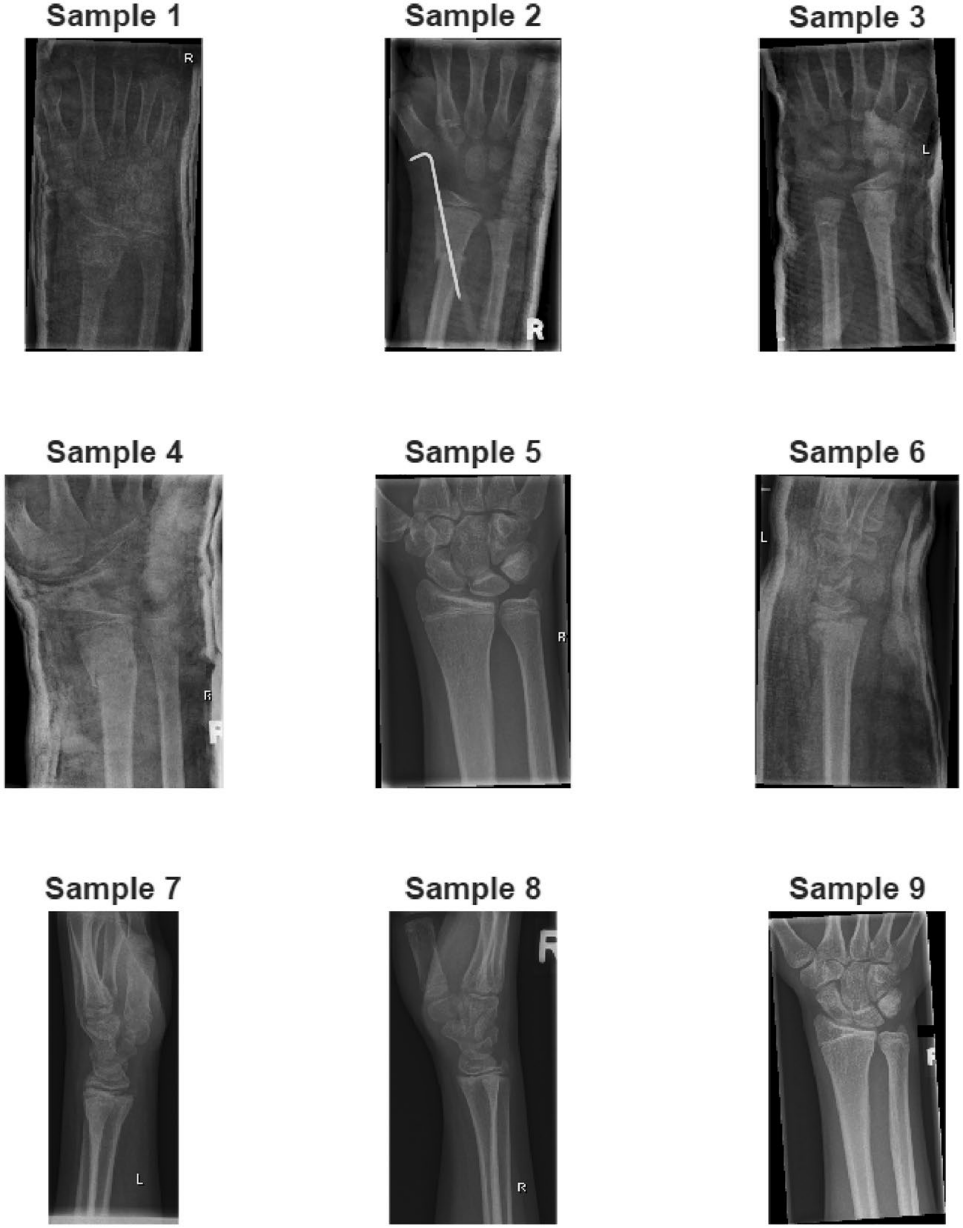
Sample images from GRAZPEDWRI-DX dataset.

The GRAZPEDWRI-DX dataset^19^ is a publicly available dataset containing approximately 20,327 composite wrist trauma X-ray images from 6,091 pediatric patients. The images are annotated and labeled by pediatric radiologists and include clinical findings such as fractures, soft tissue anomalies, and metal objects. The dataset can be used in various deep learning tasks.Sample images are shown in Fig. 4 and class sample numbers are shown in Table 2.

**Table 2:** Class distribution of GRAZPEDWRI-DX dataset.

Finding	Count
Fracture	10643
Soft tissue abnormality	3529
Metal object	1142
Periosteal reaction	977
Other abnormalities	2031
Normal	6005
Total	20237

**Fig. 5:**
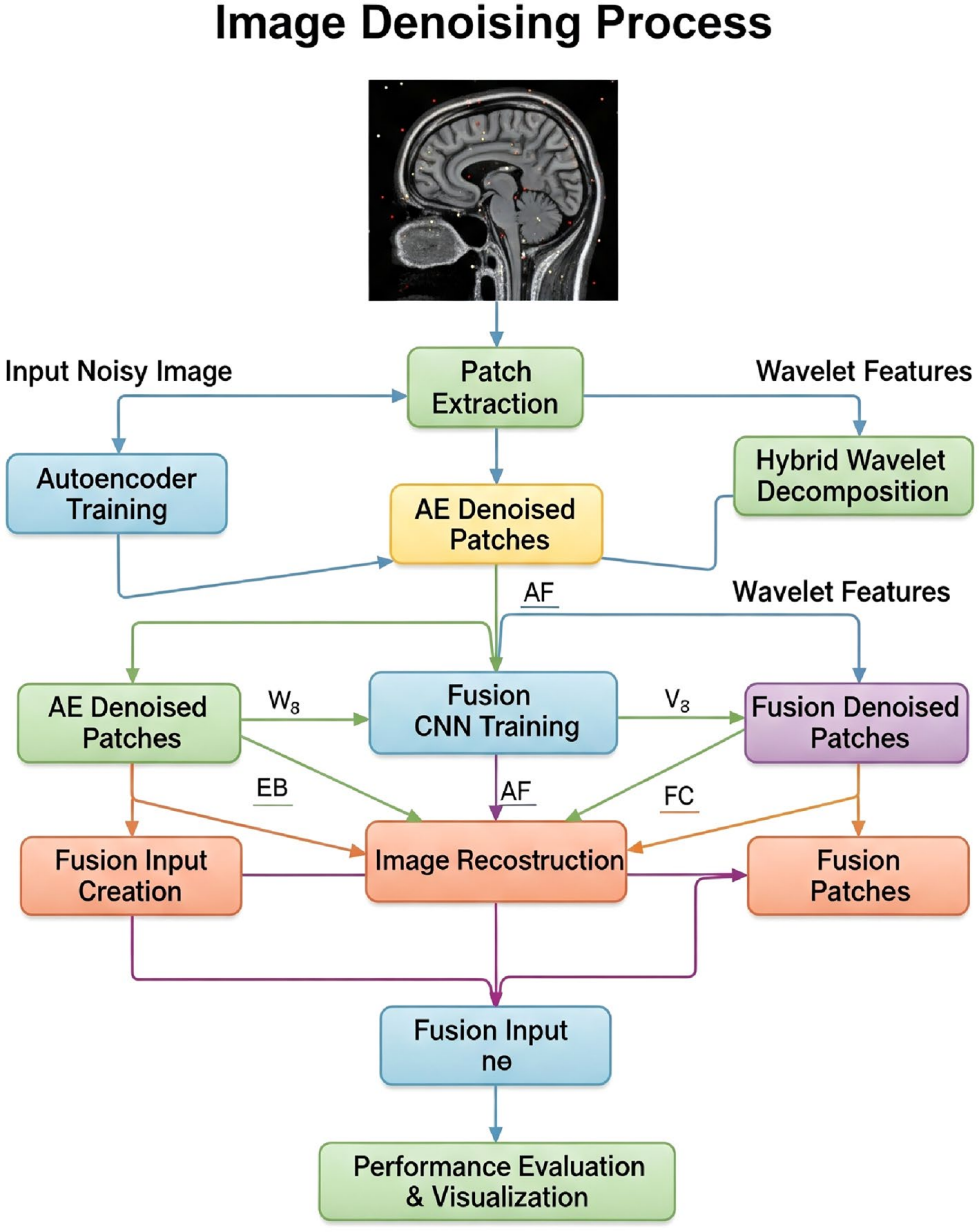
Flowchart of study.

The flow chart of this study and the algorithm of the proposed method are shown in Fig. 5 and Algorithm 1.

**Algorithm 1:**
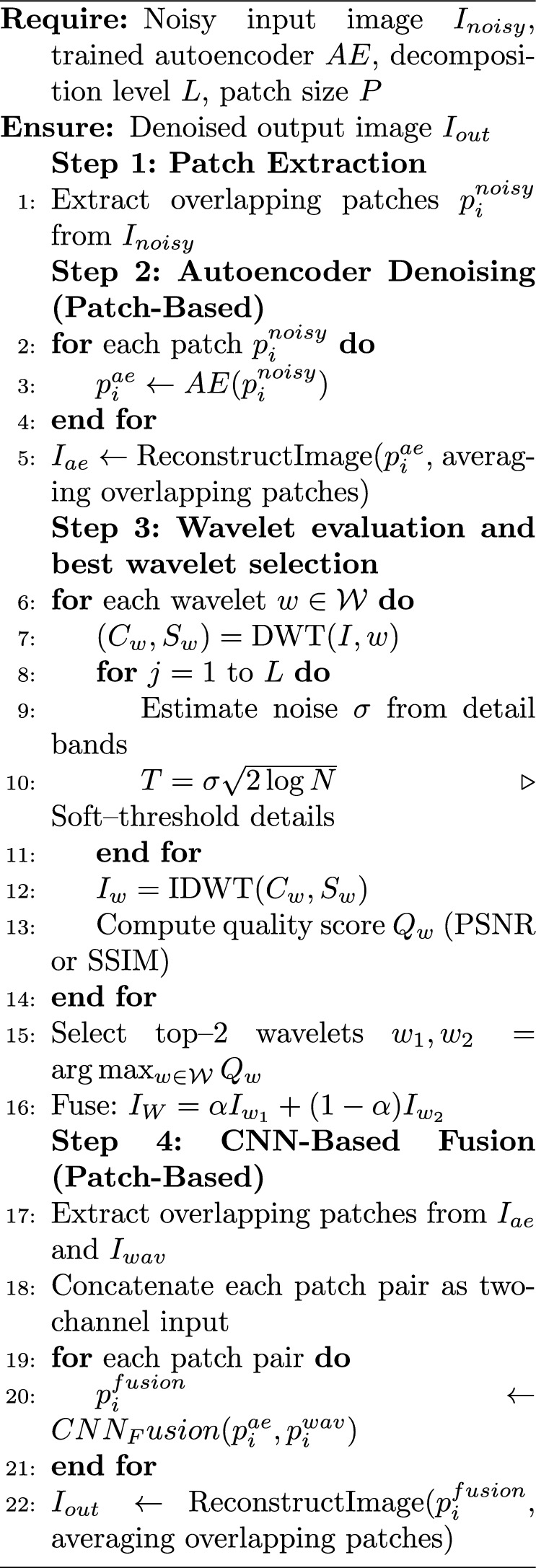
Proposed method.

### Wavelet

The wavelet transform is a method that enables multi-resolution decomposition by decomposing a signal into approximate (low-frequency) and detail (high-frequency) components. The noise targeted in this study is predominantly found in the high-frequency sub-bands. Thresholding the detail coefficients and reconstructing the signal suppress the noise while preserving structural information^20^. In this study, five widely used wavelet families (Sym4, Db4, Bior3.3, Coif2, and Haar) are used to provide a diverse representation of orthogonal, biorthogonal, and compact support filters.

Sym4 and Db4 provide orthogonal and orthogonal compact support, respectively, enabling efficient energy concentration. Bior3.3 provides symmetric, linear-phase reconstruction that improves edge preservation. Coif2 improves smoothness and feature localization by balancing compact support with vanishing moments in both scaling and wavelet functions. The Haar basic wavelet function is intended to facilitate sharp transition analysis and provide a computationally efficient basis.

The wavelet model used in this study evaluates five different wavelets instead of a fixed wavelet selection, determines the two most successful wavelets based on PSNR for each image, and performs a weighted fusion of these outputs. Thus, the method aims to increase its adaptive ability to adapt to different texture and noise characteristics.

### Autoencoder

Deep learning methods are capable of extracting deep semantic features from images, which plays a crucial role in machine learning-based diagnosis and classification processes. However, as the layer depth and complexity of the network increase, the size of the extracted features grows rapidly, posing a significant challenge in terms of dimensionality reduction and computational cost^21^. Autoencoder is an artificial neural network-based model that learns a low-dimensional representation of input data by compressing it. Autoencoder is an artificial neural network-based model designed for compressing high-dimensional data and learning important features. Its basic structure consists of two main components: an encoder and a decoder. The encoder transforms the input data from dimension ${{\bf x}} \in {\mathbb {R}}^n$ into a lower-dimensional latent representation ${{\bf z}} \in {\mathbb {R}}^m$ (usually m<n), while the decoder uses this latent representation to produce a reconstruction of dimension ${{\bf x}} \in {\mathbb {R}}^n$ in the original data space. The goal is to minimize the error function between the input and the reconstruction.

The loss function commonly used in the training process is expressed as the mean squared error (MSE) between the input and output:

3 $$\begin{aligned} {\mathcal {L}}({{\bf x}}, \hat{{{\bf x}}}) = \frac{1}{n} \sum _{i=1}^{n} (x_i - {\hat{x}}_i)^2 \end{aligned}$$

The goal of a denoising autoencoder is to build a system that can learn accurate representations despite noise added to the input. The hidden layer must be robust to small distortions in the input by capturing high-level information. Therefore, while the input to the model is noisy, the expected output is clean^22,23^.

The model’s parameters are updated using backpropagation and optimization algorithms (e.g., Adam).

Autoencoders are particularly effective in applications where data size is high and essential features need to be extracted. Autoencoder enables the learning of complex and abstract feature by using deep layers. Denoising autoencoders, adapted for noisy data, provide more robust feature extraction by deliberately adding noise to the input data and then cleaning it out.

In this study, the encoder portion of the model consists of three convolution layers, and the latent vector size was experimentally optimized. The decoder portion is structured symmetrically with the encoder and aims to successfully reconstruct the original image.

**Table 3:** Quantitative complexity comparison of the proposed framework with state of arts (SOTA) denoising methods.

Method	Conv. layers	Max filters	Params
DnCNN	17	64	$\sim$560K
FFDNet	15	64	$\sim$550K
Wavelet + CNN	12	64	$\sim$1.2M
Patch-based autoencoder	7	64	$\sim$110K
Fusion CNN (ours)	7	64	$\sim$130K
**Proposed (total)**	14	64	$\sim$240K

In Table 3, the architecture of the proposed method is compared with CNN-based and hybrid noise reduction approaches widely used in the literature. The proposed framework consists of two convolutional networks and contains approximately 240K trainable parameters in total; this value is significantly lower compared to U-Net and deep CNN-based methods with millions of parameters. Thanks to the patch-based processing strategy, memory usage and computational load are significantly reduced, while noise suppression and structural detail preservation are achieved in a balanced way through frequency-domain priors.The patch-based architecture makes the proposed method more suitable for practical applications by reducing memory usage. This structure clearly demonstrates that the proposed method is both computationally efficient and applicable clinical and research environments in practice. The fusion network, which is the distinctive component of the proposed method, combines wavelet-based multi-resolution representations with local structural information obtained from the autoencoder under a single framework, taking advantage of the complementary benefits of both approaches. This fusion mechanism preserves global information in the frequency domain during the noise suppression process while preventing the loss of fine details learned by the autoencoder in the spatial domain. Fusion mesh creates an effective balance between these two different information sources, enabling high restoration performance without the need for more complex and deeper meshes.

### Proposed method

Image fusion is an important method that enables richer and more meaningful outputs by combining information obtained from different sensors or data sources. In recent years, deep learning-based methods, particularly CNNs, have demonstrated significant success in this area. CNN-based fusion models can better learn complex patterns in data compared to traditional methods and produce more accurate predictions by effectively integrating information from different sources. This approach is used in many application areas, such as the fusion of stereo images, multi-spectral data, or information obtained from different modalities. CNNs are widely used in image fusion. CNN-based fusion methods effectively extract local features from images from different sources, successfully preserving detail and edge information. These methods can provide higher accuracy and visual quality compared to traditional pixel- or transform-based methods, especially when combining multi-focus, multi-spectral, or multi-modal data.

A new end-to-end architecture that integrates the outputs of CNNs, transformers, transforms, autoencoders, and statistical methods is being used for multi-focus image fusion. The CNN arm is responsible for learning the local features necessary to preserve fine details in focused and unfocused regions. This study proposes a CNN fusion model designed for patch-based inputs. The model is structured to process two different inputs (e.g., left and right images from stereo cameras or different spectral bands)^24^.

The imageInputLayer([patchSize 2], ’Normalization’, ’none’) used as the input layer allows for simultaneous processing of two data streams. Multiple convolutional and activation (ReLU) layers, placed sequentially, learn low-level features (edges, textures, etc.) in the input data and transform this information into more complex features. These layers are designed using 64-32-16-32-64 filters, respectively, enabling information extraction at different depths. The output layer,convolution2dLayer(3,1, ’Padding’,’same’), produces a continuous value output by combining all the acquired features into a single channel. The subsequent regressionLayer allows the model to predict continuous outputs such as pixel values instead of classification. This architecture is designed to exhibit high performance in applications such as fusion of multi-sensor data, depth map generation, or multi-spectral image analysis (in Supplementary files).

For each wavelet component $W_{\text {k}}$, its contribution weight $alpha_{\text {k}}$is defined as:

4 $$\begin{aligned} & \alpha _k = \frac{10^{\frac{\text {PSNR}_k}{10}}}{\sum _{j=1}^{K} 10^{\frac{\text {PSNR}_j}{10}}} \end{aligned}$$

5 $$\begin{aligned} & W_{\text {fused}} = \sum _{k=1}^{K} \alpha _k W_k \end{aligned}$$

The fused wavelet-enhanced patch is as follows:

6 $$\begin{aligned} W_{\text {fused}} = \sum _{k=1}^{K} \alpha _k W_k \end{aligned}$$

where $PSNR_{\text {k}}$ is the wavelet-only denoising score for wavelet type $k$. $K$ is the number of wavelet families used.

**Fig. 6:**
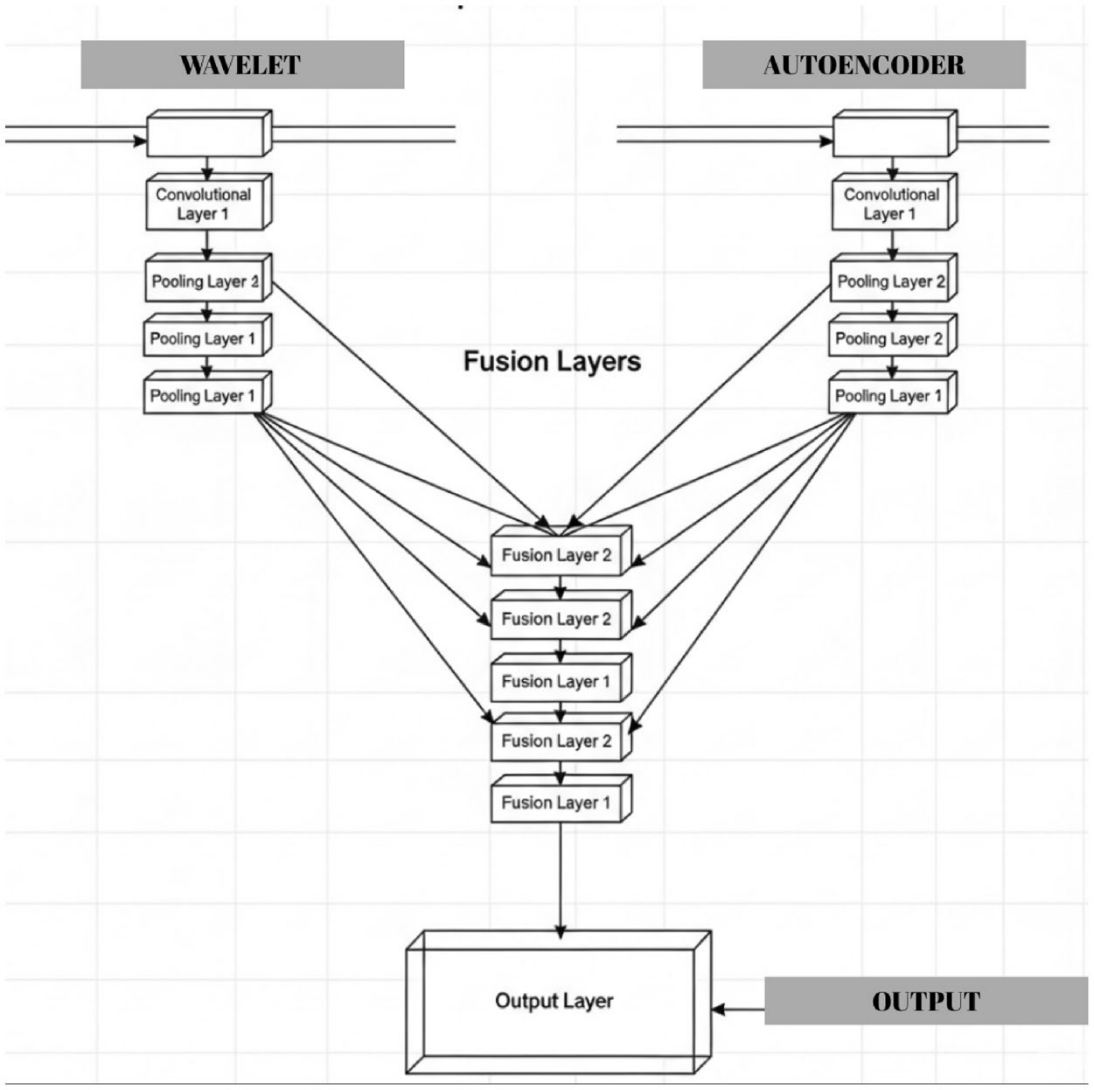
CNN fusion architecture.

Filter size, layer, and activation functions and structures are given in this section. In Figure 6 block diagram of the proposed hybrid denoising framework.

(a) Autoencoder branch: noisy fetal ultrasound images are decomposed into non-overlapping 64$\times$64 patches, which are fed into a convolutional AE composed of stacked 3$\times$3 convolution and ReLU layers to learn a clean patch reconstruction (in Table 4).

(b) Wavelet branch: the same noisy image is processed by five candidate wavelet families (Sym4, Db4, Bior3.3, Coif2, Haar) using 4-level DWT, coefficient thresholding, and reconstruction; PSNR is computed for each wavelet, and the two best outputs are fused into a single hybrid wavelet image using PSNR-proportional weights.

(c) Fusion CNN: for each spatial location, a 2-channel input patch is formed by stacking the AE output and the hybrid wavelet patch. These patches are then passed through a shallow CNN with successive 3$\times$3 convolution + ReLU layers and a regression output layer to generate the final denoised image (in Table 5).

**Table 4:** Proposed autoencoder architecture for patch-based denoising.

Stage	Layer	Filters/units	Kernel/stride	Activation
Encoder	Conv2D	64	$3\times 3$	ReLU
	Conv2D	32	$3\times 3$	ReLU
	Conv2D	16	$3\times 3$	ReLU
Decoder	Conv2D	32	$3\times 3$	ReLU
	Conv2D	64	$3\times 3$	ReLU
	Conv2D (output)	1	$3\times 3$	Linear

**Table 5:** CNN fusion network architecture (AE + hybrid wavelet input).

Stage	Layer	Filters	Kernel	Activation
Fusion CNN	Input ($64\times 64\times 2$)	–	–	–
	Conv2D	64	$3\times 3$	ReLU
	Conv2D	32	$3\times 3$	ReLU
	Conv2D	16	$3\times 3$	ReLU
	Conv2D	32	$3\times 3$	ReLU
	Conv2D	64	$3\times 3$	ReLU
	Conv2D (output)	1	$3\times 3$	Linear

The success of the model depends not only on the architectural design but also on the training of the AE and CNN fusion components with appropriate hyperparameters. The correct selection of settings such as optimizer, number of epochs, and loss function directly affects both convergence stability and noise reduction performance. Therefore, the training hyperparameters used are summarized in Table 6.

**Table 6:** Training hyperparameters for AE and fusion models.

Parameter	Autoencoder	Fusion CNN
Patch size	$64\times 64$	$64\times 64$
Optimizer	Adam	Adam
Loss function	MSE	MSE
Epochs	10	10
Batch size	128	128

When the studies in the literature are examined methodologically, image noise reduction methods are generally grouped into 4 categories: wavelet-based filtering methods, autoencoder-based deep learning models, purely CNN-based architectures, and hybrid methods that combine different components of these approaches. While each category offers certain advantages, it also has limitations such as loss of structural detail, over-smoothing, susceptibility to speckle-type noise, or dependence on fixed transformations. The Table 7 below systematically summarizes the basic characteristics of these method groups and outlines which shortcomings of existing techniques the proposed Wavelet–AE–CNN fusion approach addresses.

**Table 7:** Comparison of existing method categories with the proposed hybrid Wavelet–AE–CNN approach.

Method category	Strengths	Limitations	How the proposed method improves
Wavelet-based methods^25–28^	Strong noise suppression, multi-resolution analysis	Oversmoothing, loss of high-frequency detail, fixed transform	Adaptive multi-wavelet fusion, preserves structure and texture
Autoencoder-based methods^7,23,29–31^	Learns nonlinear noise mapping	Risk of learning noise as texture, blur artifacts	Combines AE with wavelet outputs to avoid over-smoothing
CNN-based methods^6,24,32,33^	High spatial feature learning capacity	Produces overly smooth outputs, struggles with speckle noise	Wavelet priors reintroduce high-frequency anatomical details
Hybrid wavelet-DL methods^34–37^	Improved edge preservation	Mostly fixed fusion rules, limited adaptivity	PSNR-weighted fusion + CNN provides learned nonlinear integration
Proposed method	Integrates AE, multi-wavelet, CNN fusion adaptively	The limitations of the proposed method are elaborated in the Limitations section	Best balance of detail preservation, noise suppression, and stability

In this study, the train-test division was performed entirely at image level, and patch extraction was applied only after this separation to prevent data leakage in the patch-based learning process.The calculation was made by considering the average values obtained from all test samples during the testing phase. Non-overlapping patches were used in the patch-based extraction and reconstruction process; therefore, no overlapping-averaging or weighted merging operations were applied. The results obtained within this evaluation framework clearly demonstrate that the proposed method exhibits consistent and superior performance under different datasets and noise types, and especially achieves a balanced success between noise suppression and the preservation of structural/anatomical details.

### Sensitivity analysis of key design parameters

The architectural configuration and training hyperparameters summarized in Tables 6 were determined through pre-selection experiments rather than a comprehensive optimization process. Key design parameters such as patch size, wavelet decomposition level, number of candidate wavelet families, and CNN depth were evaluated independently while other settings were kept constant.

32$\times$32, 64$\times$64, and 128$\times$128 patch sizes were examined. Smaller patches failed to adequately represent anatomical context, while larger patches reduced the number of effective training instances, leading to a decrease in generalization performance. The 64$\times$64 patch size consistently provided the most balanced result between local detail preservation and global structural consistency.

Similarly, wavelet decomposition levels ranging from 3 to 5 were tested. Lower levels were insufficient for suppressing high-frequency noise, while higher levels caused slight oversmoothing. Level 4 offered the most stable performance across different datasets and noise types. Increasing the number of candidate wavelet families resulted in limited performance gains but significantly increased computational cost. Finally, deeper CNN fusion architectures were also evaluated; however, the marginal performance improvements obtained did not offer a preferable advantage considering the increased training complexity and inference time.

## Results and discussion

This section presents experimental results obtained from the proposed image restoration framework. The evaluation is performed on medical images affected by Gaussian and Speckle noise and passed through the designed hybrid denoising and reconstruction pipeline. Performance is evaluated using SSIM and PSNR values. Furthermore, visual comparisons of the restored images are presented to demonstrate the effectiveness of the proposed approach compared to traditional statistical filtering and state-of-the-art deep learning methods. In our framework, the contribution of the wavelet stage is governed by a strictly data-driven selection process rather than predefined theoretical preferences. For each image, all five candidate wavelet families are independently evaluated through the same multi-level decomposition–thresholding–reconstruction pipeline, and their denoising performance is quantitatively assessed using PSNR. The two highest-performing wavelets are then fused using a proportional weighting strategy, allowing the model to adapt its representation to the specific statistical characteristics of the input. This dynamic evaluation not only reveals the practical strengths of different wavelet families under varying noise and anatomical conditions, but also ensures that the hybrid pipeline benefits from the most effective combination at each instance. Consequently, the wavelet stage contributes both analytically and empirically, reinforcing the robustness and robustness of the proposed denoising approach. The proposed autoencoder consists of a symmetric 6-layer convolutional structure with 64–32–16 filters in the encoder and 32–64–1 filters in the decoder. All convolution kernels are 3$\times$3 with ReLU activations (linear in output). Models were trained for 10 epochs with Adam (batch size 128).

In this study, the proposed hybrid method was compared with the classical Wiener filter on different noise types and different datasets.The experimental results in this study were obtained by dividing the input images into 64x64 patches. To determine the appropriate patch size for training the model, preliminary experiments were conducted with 32$\times$32, 64$\times$64, and 128$\times$128 sizes. The 32$\times$32 patch size did not adequately represent anatomical structures, leading to a disruption of edge continuity and a decrease in SSIM values. In contrast, the 128$\times$128 patch size reduced the number of removable samples and increased the computational load; it also produced lower PSNR/SSIM results due to a tendency towards overfitting. As a result of the experiments, the 64$\times$64 patch size showed the most balanced performance in terms of both detail preservation and structural integrity, and this size was used in all experiments.The patch-based approach not only reduces computational overhead but also enables the capture of more localized and detailed features during the learning process. Specifically, in evaluating denoising performance, it was observed that the 64x64 patch-based processing method produces more stable and reliable results in the SSIM and PSNR metrics.

**Fig. 7:**
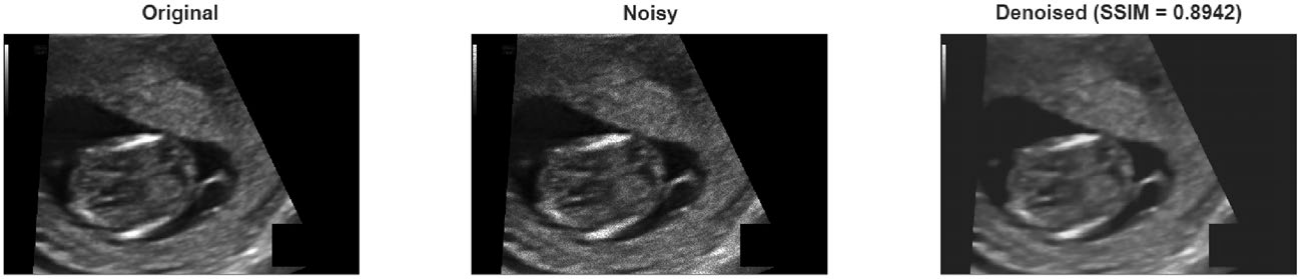
A sample result from fetal ultrasound dataset with Gaussian noise.

**Fig. 8:**
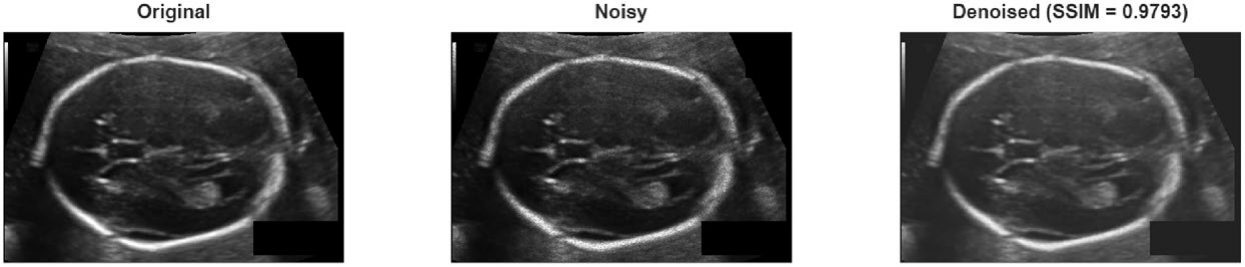
A sample result from fetal ultrasound dataset with Speckle noise.

The results show that the proposed method is significantly superior in terms of PSNR and SSIM. Experiments on the fetal ultrasound dataset revealed that the proposed method is highly effective against both Gaussian and Speckle noise. In the case of Gaussian noise, improvements in PSNR of approximately 5–11 dB and SSIM of up to 0.30 were achieved compared to the Wiener filter. The results obtained for Speckle noise are even more striking; at low noise levels, the proposed method achieved a PSNR of 48.12 dB and an SSIM of 0.99, yielding results almost comparable to the original, pristine image quality (in Table 8). This suggests that the proposed method could be clinically significant, especially in modalities susceptible to speckle noise, such as ultrasound. Figure 7 shows the result obtained after applying Gaussian noise to the sample taken from fetal ultrasound dataset, and Fig. 8 shows the result obtained after applying speckle noise.

**Table 8:** Denoising comparison under Gaussian and Speckle noise on fetal ultrasound dataset.

Noise level	Gaussian (PSNR/SSIM)			Speckle (PSNR/SSIM)		
	Noisy	Wiener	Proposed	Noisy	Wiener	Proposed
Low	26.94/0.28	33.38/0.54	39.18/0.95	42.74/0.97	43.53/0.98	48.12/0.99
Medium	17.43/0.06	24.43/0.42	35.74/0.84	32.74/0.85	34.81/0.95	40.87/0.98
High	12.61/0.03	18.26/0.24	25.27/0.65	27.62/0.76	29.65/0.90	37.40/0.96

**Fig. 9:**
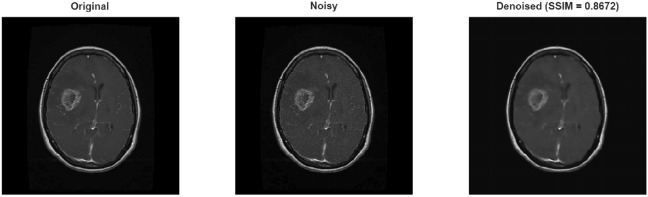
A sample result from brain tumor dataset with Gaussian noise.

**Fig. 10:**
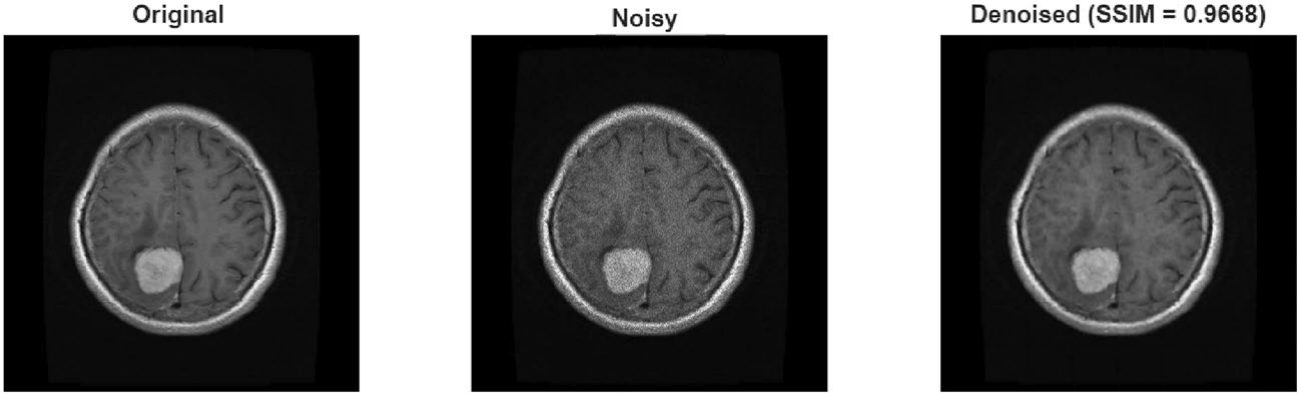
A sample result from brain tumor dataset with Speckle noise.

**Fig. 11:**
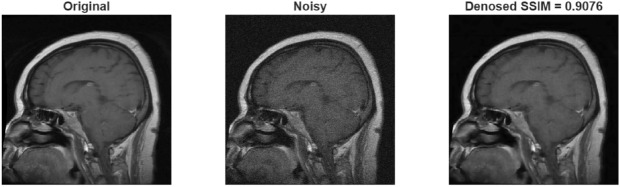
A sample result from brain tumor dataset with Rician noise.

Experiments on the brain tumor dataset also exhibited similar trends. In the results obtained for Gaussian noise, the Wiener filter produced slightly higher PSNR values than the proposed method at low noise levels (32.28 dB vs. 29.62 dB). However, when examining the SSIM values under the same conditions, it is seen that the proposed method reaches a significantly higher value of 0.91 and preserves structural integrity much better (in Table .This finding demonstrates that the proposed method prioritizes structural and visual quality over pure numerical improvement, thus offering a more significant advantage for medical imaging. At medium and high noise levels, the proposed method demonstrated a clear superiority in both PSNR and SSIM. The results obtained for speckle noise also show that the proposed method outperformed the Wiener filter at all levels. Speckle noise, in particular, yielded an increase in PSNR of approximately 7 dB and SSIM of 0.12 dB (in Table 9). Figure 9 shows the result obtained after applying Gaussian noise to the sample taken from brain tumor dataset, and Figs. 10 and 11 shows the result obtained after applying speckle and rician noises,respectively.

**Table 9:** Denoising comparison under Gaussian, Speckle, and Rician noise on brain tumor dataset.

Noise level	Gaussian (PSNR/SSIM)			Speckle (PSNR/SSIM)			Rician (PSNR/SSIM)		
	Noisy	Wiener	Proposed	Noisy	Wiener	Proposed	Noisy	Wiener	Proposed
Low	26.25/0.42	32.28/0.75	29.62/0.91	37.49/0.94	37.36/0.95	41.21/0.98	26.99/0.34	32.45/0.64	29.00/0.90
Medium	16.01/0.11	23.51/0.40	30.61/0.78	27.53/0.76	31.38/0.90	35.51/0.95	17.02/0.10	21.70/0.32	27.11/0.59
High	11.02/0.06	18.85/0.24	27.27/0.67	22.38/0.62	26.50/0.81	33.92/0.93	16.42/0.02	17.90/0.11	22.17/0.47

The proposed method demonstrated higher accuracy in noise suppression compared to the Wiener filter for both Gaussian and Speckle noise. It holds the potential to improve image quality by preserving diagnostically important anatomical structures, particularly in medical applications such as ultrasound and brain imaging.

**Fig. 12:**
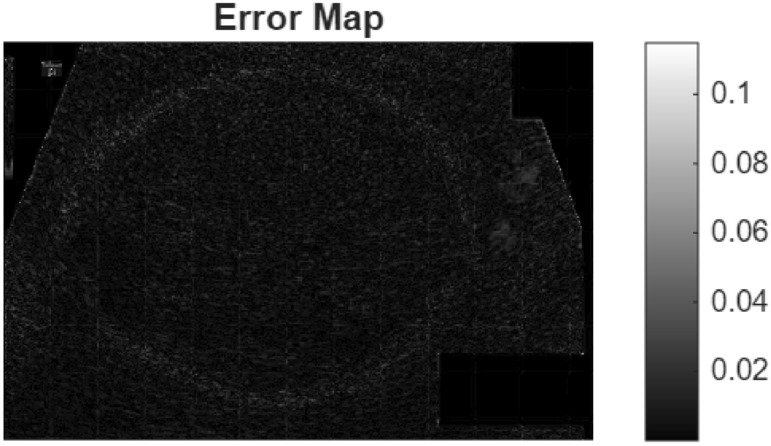
Difference image of fetal dataset results for speckle noise.

Examining the difference image in the figure, the structural edges in the reconstructed brain scan remain highly consistent with the ground truth. Small discrepancies are concentrated in fine-textured regions, while key anatomical boundaries are preserved. This behavior is consistent with the reported SSIM, indicating strong structural accuracy.(in Fig. 12)

**Fig. 13:**
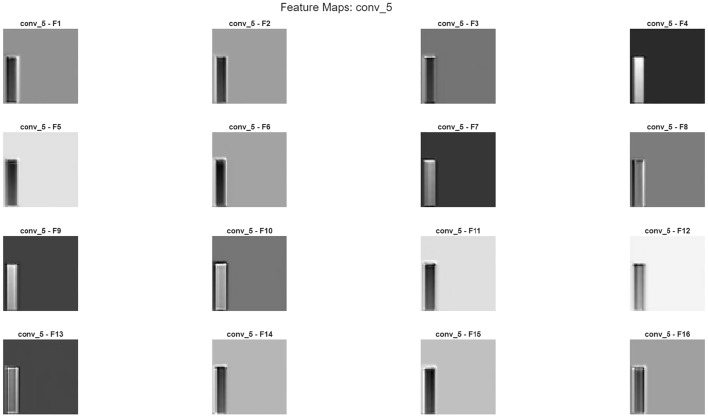
Feature map a sample patch from brain tumor dataset conv5.

**Fig. 14:**
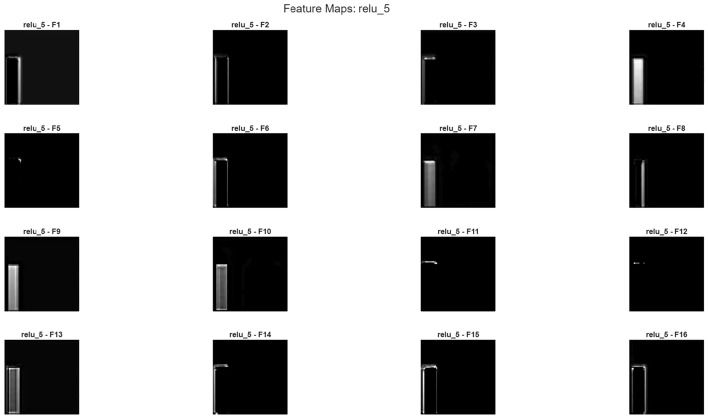
Feature map a sample patch from brain tumor dataset conv5(continue).

**Fig. 15:**
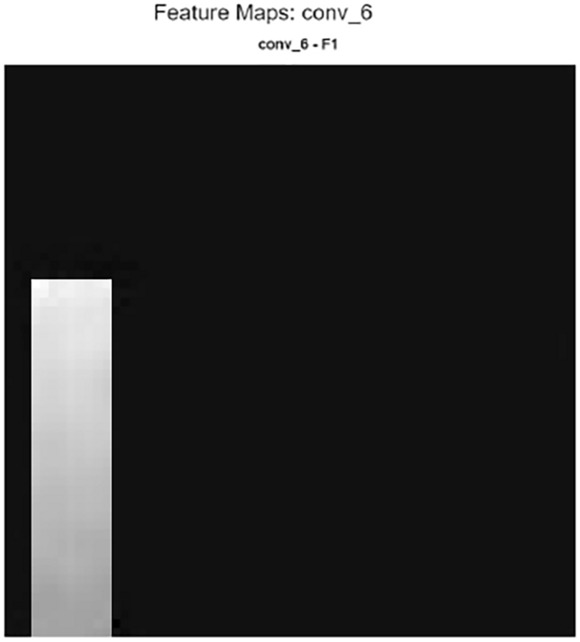
Feature map a sample patch from brain tumor dataset conv6.

Furthermore, it is possible to evaluate the model’s noise reduction performance not only with quantitative metrics such as PSNR and SSIM, but also qualitatively through internal layer activations. In particular, each of the three main components of the hybrid structure-wavelet transform, autoencoder, and CNN fusion layer-exhibits different levels of sensitivity to structural, textural, and frequency components. Therefore, examining the feature maps generated in the intermediate layers visually reveals in which regions the model relies on wavelet representation, in which regions on the global context of the autoencoder, and at which stage on the deep spatial features of the CNN.Feature maps are shown in Figs. 13, 14 and 15

**Table 10:** Runtime comparison (training + inference) for different datasets and noise models.

Noise type	Dataset	Training time	Inference time
Gaussian	Fetal ultrasound	75 min	58 sec
	Brain tumor MRI	82 min	57 sec
Speckle	Fetal ultrasound	41 min	23 sec
	Brain tumor MRI	52 min	79 sec
Rician	Brain tumor MRI	100 min	79 sec

When Table 10 is examined, it is seen that the denoising operations performed under Gaussian noise take longer than under speckle noise. This shows that Gaussian distortion puts more strain on the model learning dynamics. However, due to the approximately two-fold difference between the sample numbers in the datasets and the corresponding increase in the number of patches, the analyses performed on the brain tumor dataset appeared to take longer.It should also be noted that the reported inference times correspond to full-image reconstruction using a patch-based pipeline. Given the shallow CNN fusion architecture and the possibility of parallel patch processing, the obtained inference times indicate that the proposed method is suitable for offline and near–real-time pre-processing scenarios in clinical workflows.

The experimental findings presented in this section demonstrate that the proposed method outperforms the Wiener filter across various noise types and datasets. The significant increase in SSIM values, in particular, demonstrates the method’s strong advantage in preserving structural information. A comparison of the results from a clinical and academic perspective is presented in Table 11.The highest mean values obtained in this study are shown in the table.

**Table 11:** Comparison of recent wavelet, autoencoder, and CNN-based image denoising studies (2022–2025).

Year	Author(s)	Method	Performance (PSNR/SSIM)
2022	Tian et al.^25^	Multi-stage CNN with wavelet transform (MWDCNN)	32.5 dB / 0.91
2023	Shi et al.^38^	Ultrasound image denoising autoencoder with lightweight attention	32.9 dB / 0.91
2023	Abuya et al.^34^	Wavelet-anisotropic Gaussian filter-based CNN for CT images	33.1 dB / 0.92
2023	Liang et al.^39^	ResWCAE: Residual wavelet-conditioned autoencoder for biometric images	32.8 dB / 0.91
2024	Zhou et al.^40^	AE-based LAD-CNN with lightweight attention blocks	32.9 dB / 0.91
2025	Fan et al.^35^	Wavelet-domain frequency-mixing transformer unfolding network for LDCT	34.0 dB / 0.94
2025	Malyugina et al.^41^	Wavelet-based topological loss for low-light image denoising	34.2 dB / 0.94
2025	Proposed Method	Wavelet-Enhanced Autoencoder Based Image Denoising with CNN Fusion	48.12dB / 0.99

The performance of recent image denoising methods combining wavelet transforms, autoencoders, and convolutional neural networks has significantly improved over the last five years. Studies such as MWDCNN^25^ and Haar-DWT + DnCNN ensembles^34^ demonstrate that multi-stage or hybrid wavelet-based approaches can effectively enhance PSNR and SSIM metrics. Residual wavelet-conditioned autoencoders^39^ and AE-based LAD-CNN models^40^ show comparable improvements, particularly in medical imaging applications. More recent works leveraging wavelet-domain transformer unfolding^35^ and wavelet-derived topological loss^41^ achieve the highest reported PSNR and SSIM values, indicating that integrating frequency-domain information with deep learning architectures leads to superior denoising performance.

The proposed method significantly improved denoising performance in both fetal ultrasound and brain tumor images, providing a strong advantage in preserving structural details, particularly with increased SSIM values. This is critical for the accurate interpretation of images in clinical diagnostic processes. While higher PSNR does not always guarantee better perceptual quality, increased SSIM indicates the preservation of directly visible details.

While PSNR and SSIM gains in previous studies are incremental, this is significant given that traditional wavelet-based and CNN-only approaches tend to reach saturation near approximately the 35dB PSNR in similar environments. The proposed approach addresses this performance limitation by integrating frequency-domain sparsity with spatial feature learning, providing more effective edge preservation and noise suppression beyond the limitations of previous methods.

The proposed method was evaluated not only based on average PSNR and SSIM values, but also in terms of statistical variability. Accordingly, standard deviations and 95% confidence intervals were calculated for the performance metrics obtained in the test sections of both datasets, thus measuring the consistency of the model across different samples. Total sample number for these calculations is equal to test sample number for each dataset. This additional analysis is important in demonstrating the overall stability and robustness of the method against data variation. The results are presented in Table 12.

**Table 12:** Statistical robustness analysis of the proposed method under Gaussian and Speckle noise. Mean, standard deviation (Std), and 95% confidence intervals (CI) are reported for PSNR and SSIM.

Dataset / noise	PSNR mean	PSNR std	95% CI (PSNR)	SSIM mean	SSIM std	95% CI (SSIM)
Fetal ultrasound / Gaussian	38.60	1.23	[38.45 – 38.75]	0.9474	0.0191	[0.9450 – 0.9498]
Brain Tumor MRI / Gaussian	35.30	2.83	[34.59 – 36.02]	0.9093	0.0228	[0.9035 – 0.9150]
Fetal ultrasound / Speckle	42.13	4.68	[36.84 – 47.42]	0.9767	0.0153	[0.9590 – 0.9940]
Brain Tumor MRI / Speckle	36.88	3.87	[32.51 – 41.25]	0.9533	0.0205	[0.9301 – 0.9761]
Brain tumor MRI / Rician	32.80	2.77	[30.92 – 40.48]	0.8599	0.0356	[0.8160 – 0.8823]

**Table 13:** Statistical robustness analysis of the proposed method under Gaussian and Speckle noise using PSNR–SSIM–based wavelet selection. Mean, standard deviation (Std), and 95% confidence intervals (CI) are reported for PSNR and SSIM.

Dataset / noise	PSNR mean	PSNR std	95% CI (PSNR)	SSIM mean	SSIM std	95% CI (SSIM)
Fetal ultrasound / Gaussian	38.88	2.12	[38.54–38.99]	0.9494	0.0172	[0.9460–0.9502]
Brain tumor MRI / Gaussian	35.58	3.09	[34.80–36.36]	0.9124	0.0216	[0.9069–0.9178]
Fetal ultrasound / Speckle	42.44	4.88	[36.99–47.65]	0.9787	0.0132	[0.9600–0.9960]
Brain tumor MRI / Speckle	37.01	4.07	[32.71–41.99]	0.9600	0.0201	[0.9388–0.9701]
Brain tumor MRI / Rician	34.80	3.98	[31.7240.88]	0.8607	0.0356	[0.8190–0.8923]

Although PSNR has been widely used as an objective metric for wavelet selection in image denoising, it is well known that PSNR alone may not fully capture perceptual and structural fidelity, particularly in medical imaging applications. To address this limitation, the proposed framework was extended to incorporate SSIM alongside PSNR with equal weighting during the wavelet selection and fusion stage. Experimental observations indicate that this modification does not significantly alter the average PSNR and SSIM values, which primarily reflect overall denoising performance. However, a notable improvement is observed in terms of statistical stability and structural consistency, as evidenced by reduced variance and narrower confidence intervals in Table 13. This shows that the combined PSNR–SSIM selection strategy primarily enhances the robustness and reliability of the reconstruction rather than inflating the average performance metrics, which is particularly relevant for clinically oriented image enhancement tasks where consistency and structural reliability across samples are critical.

In addition to Gaussian and speckle noise, Rician noise was included in the evaluation to better reflect realistic noise characteristics encountered in MRI acquisitions. As shown in Table13, the proposed method exhibits a noticeable performance degradation under Rician noise compared to Gaussian and speckle cases, with PSNR and SSIM values of 34.80 dB and 0.8607, respectively. This behavior is expected due to the signal-dependent and non-zero mean nature of Rician noise, which poses a more challenging restoration problem. Nevertheless, the relatively moderate standard deviation and tight confidence interval indicate that the proposed framework maintains stable and consistent performance even under this more complex noise model. While PSNR–SSIM–based wavelet selection consistently improves the average reconstruction quality under Gaussian and speckle noise, its impact under Rician noise is more moderate, particularly in terms of SSIM. As reflected by the increase in the standard deviation in Table 13, the adaptive selection strategy produces substantial improvements for certain samples, while the signal-dependent nature of Rician noise limits structural similarity gains in others. This behavior is expected and highlights the challenging characteristics of Rician noise in MRI reconstruction.

**Fig. 16:**
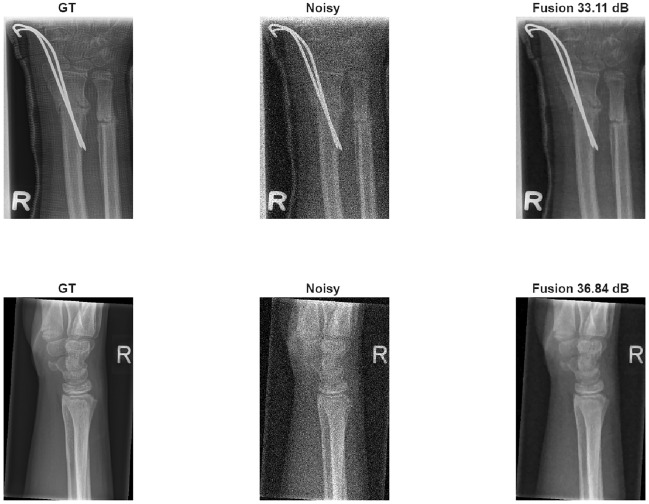
GRAZPEDWRI-DX X-ray dataset sample result by using synthetic noise.

In this study, the proposed approach was evaluated on the pediatric trauma wrist trauma X-ray dataset to examine its effectiveness under real clinical imaging conditions. This dataset consists of publicly available radiographic images with inherent noise characteristics. Additional experiments were conducted on the GRAZPEDWRI-DX wrist X-ray dataset to evaluate the performance of the proposed method on real clinical images. First, to enable the use of reference quality metrics, different levels of Gaussian and Poisson noise were synthetically added to the images in the dataset.Sample result images are shown in Fig. 16 Under these conditions, the proposed method was evaluated using reference image quality metrics such as PSNR and SSIM in Table 14.

**Table 14:** Statistical robustness analysis on the GRAZPEDWRI-DX X-ray dataset under Gaussian and Poisson noise levels. Mean, standard deviation (Std), and 95% confidence intervals (CI) are reported for PSNR and SSIM.

Dataset / noise	PSNR mean	PSNR std	95% CI (PSNR)	SSIM mean	SSIM std	**95% CI (SSIM)**
X-ray / Gaussian-Low	33.18	1.27	[32.83 – 33.53]	0.9126	0.0189	[0.9074 – 0.9178]
X-ray / Gaussian-Medium	30.42	1.94	[29.88 – 30.96]	0.8841	0.0226	[0.8778 – 0.8904]
X-ray / Gaussian-High	28.63	2.31	[27.99 – 29.27]	0.8517	0.0278	[0.8439 – 0.8595]
X-ray / Poisson-Low	31.84	1.56	[31.41 – 32.27]	0.8935	0.0207	[0.8877 – 0.8993]
X-ray / Poisson-Medium	29.27	2.04	[28.70 – 29.84]	0.8642	0.0249	[0.8572 – 0.8712]
X-ray / Poisson-High	27.11	2.58	[26.39 – 27.83]	0.8234	0.0296	[0.8151 – 0.8317]

**Fig. 17:**
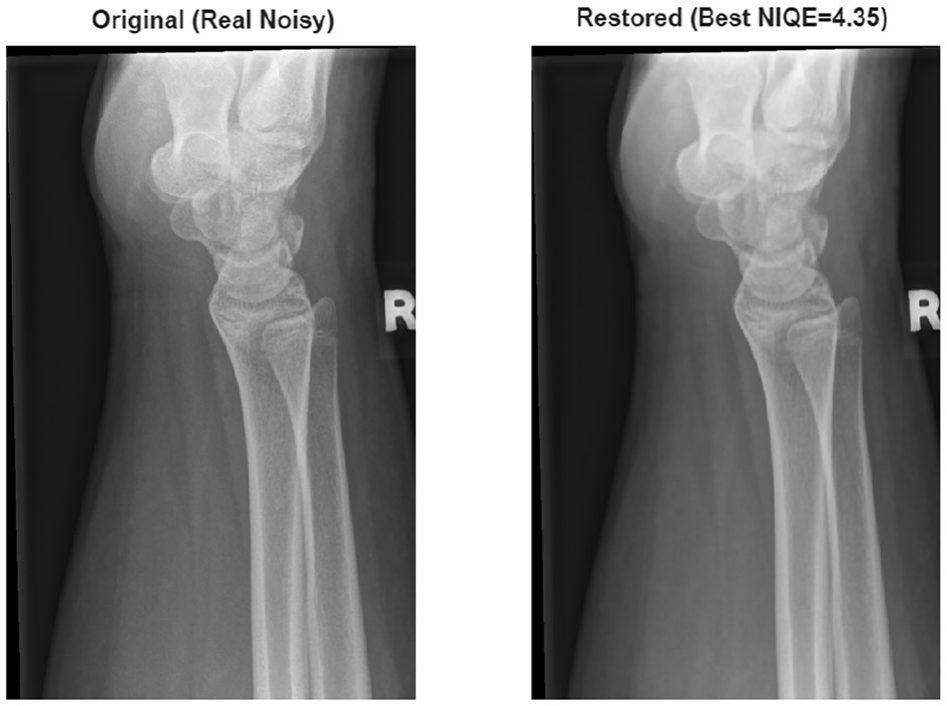
GRAZPEDWRI-DX X-ray dataset sample result.

**Fig. 18:**
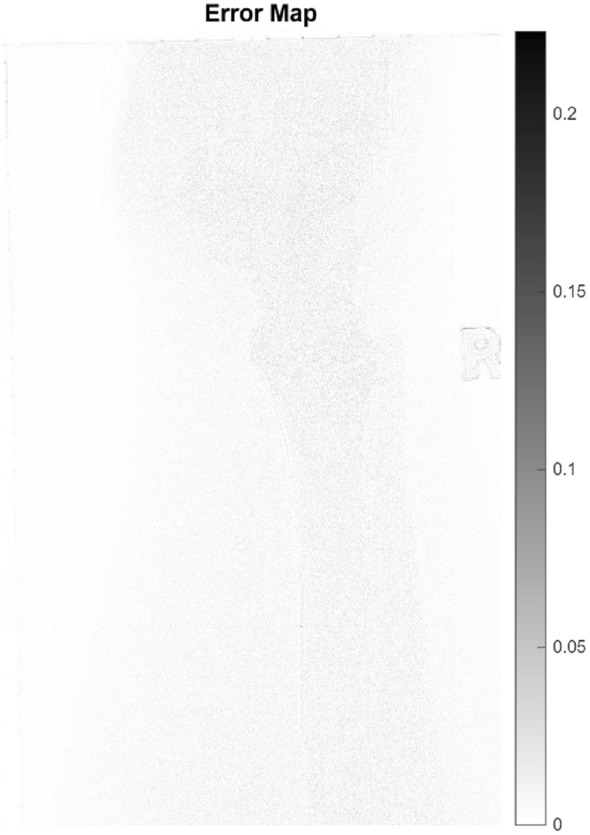
Error map for the GRAZPEDWRI-DX X-ray dataset sample.

Table 14 summarizes the performance of the proposed method on different levels of Gaussian and Poisson noise types on the GRAZPEDWRI-DX X-ray dataset. Examining the results, it is observed that increasing the noise level naturally leads to increased difficulty in the restoration problem.When Gaussian noise is applied, the PSNR values are observed to range approximately 28.63–33.18 dB, while the SSIM values range from 0.8517–0.9126. With Poisson noise, the PSNR is approximately 27.11–31.84 dB, and the SSIM is 0.8234–0.8935. It is seen that Poisson noise is more difficult to restore compared to Gaussian noise due to its structure stemming from photon count statistics, and therefore the obtained PSNR and SSIM values are relatively lower. Despite these data-related limitations, the fact that the proposed method exhibits consistent performance at all noise levels and demonstrates good performance in noise reduction in X-ray images contributes to the robustness of the study. In addition, experiments were also conducted on the original versions of the dataset to examine the effectiveness of the proposed method on real clinical images. In this case, since clean reference images were not available, the evaluation was performed using non-referenced image quality metrics. The results show that the proposed method is effective in improving image quality both under controlled conditions containing synthetic noise and on real clinical images. The NIQE and SSEQ metrics were used for analysis; lower values indicate better perceptual quality. Experimental findings demonstrate that the hybrid strategy successfully reduces spatial artifacts while preserving fracture-related anatomical edges. A statistical summary of test set is reported in Table 15, and representative visual results are shown in Figure 17 and Figure 18. The results confirm that combining classical wavelet prioritization with deep learning-based reconstruction, as in the other two datasets, provides a consistent improvement in pediatric bone image analysis compared to single-stage approaches. The robustness of the study to clinical conditions was also investigated.

For image noise reduction problems, the Denoising Convolutional Neural Network(DnCNN) residual noise learning strategy, a CNN-based method, provides effective results at different noise levels in terms of flexibility and noise level adaptation, as does FFDNet FFDNet (Fast and Flexible Denoising Network). Instead of directly predicting the clean image from the input image, DnCNN aims to learn the noise component and remove it from the input. This residual learning approach enables faster convergence of the network and reduces the gradient fading problem encountered in deep architectures. In addition, the use of batch normalization and ReLU activation functions increases the training stability of DnCNN, demonstrating its success with different noise types^42–44^. The difference between FFDNet and DnCNN is that FFDNet includes a noise level map in addition to the input image at the network input. In this way, the need to train the network separately for different noise densities with a single model is eliminated, and a significant advantage is provided in practical applications. FFDNet reduces computational complexity by processing the image through a down-sampled representation and offers fast inference even in high-resolution images.Therefore, it is thought that validating the proposed method using SOTA methods will contribute to the robustness of the study. Tables 15 and 16 compare the performance of the proposed method on the GRAZPEDWRI-DX dataset using non-referenced image quality metrics. In Table 15, the proposed method uses DnCNN and FFDNet methods asstate-of-the-art (SOTA). Considering that lower values in the NIQE and SSEQ metrics represent better perceptual image quality, the proposed method appears to achieve better results in both metrics. The proposed method’s NIQE value of 3.12 ± 0.36 shows shows a clear improvement over SOTA methods (DnCNN 4.85 ± 0.42 and FFDNet 4.61 ± 0.43 values). Similarly, the SSEQ metric value of 0.26 ± 0.08 is lower than the DnCNN (0.78 ± 0.09) and FFDNet (0.61 ± 0.10) results, demonstrating the success of the proposed method in terms of perceptual quality.

Table 16 shows the statistical robustness of the proposed method. The mean value of 3.12 and standard deviation of 0.36 obtained for the NIQE metric confirm that the model outputs are consistent across test images. The confidence interval [2.41, 3.83] indicates that the results do not show excessive fluctuation across different images and that the method has stable performance. The mean value of 0.26 and standard deviation of 0.08 obtained for the SSEQ metric support the fact that spectral and spatial features are preserved in a balanced manner during the restoration process, and the confidence interval [0.10, 0.42] supports that the proposed method produces stable results across different images. In conclusion, it is seen that the proposed method provides superior performance in terms of perceptual quality and offers stable and reliable restoration capability in real clinical X-ray images. Overall, the table shows that the deep learning-based model provides a stable quality improvement on the existing noise characteristics in the dataset; the fact that two different no-reference metrics produce results in the same direction indicates that the method can be reliably interpreted within the context of the discussion. The obtained quantitative performance values reflect the characteristics of real pediatric radiographs, not an artificially generated environment. Therefore, the results are entirely derived from real-world data conditions. These findings demonstrate that the proposed approach improves the suitability of pediatric bone images for analysis and provides a foundation for further studies.

**Table 15:** Proposed method comparison with SOTA methods by using using no-reference quality metrics.

Method	NIQE $\downarrow$	SSEQ $\downarrow$
DnCNN	4.85 ± 0.42	0.78 ± 0.09
FFDNet	4.61 ± 0.43	0.61 ± 0.10
Proposed method	3.12 ± 0.36	0.26 ± 0.08

According to the results in Tables 15, 16 and 17 obtained from analyses performed using SOTA methods, the proposed method demonstrates a significant advantage in both image quality and computational efficiency. Statistical robustness analysis reveals narrow confidence intervals for both metrics, confirming the method’s stable performance across different test samples. In terms of computational complexity, the proposed approach offers a lighter structure compared to DnCNN and FFDNet, with fewer parameters, lower FLOPs, and more limited peak memory usage. This can be explained by performing part of the noise suppression process in the wavelet space and running the deep mesh components on more distinctive and simplified features. The proposed method not only improves quality but also offers a practical and scalable solution for use in resource-constrained hardware.

**Table 16:** Statistical robustness analysis of the proposed method on the GRAZPEDWRI-DX test set. Mean, standard deviation (Std), and 95% confidence intervals (CI) are reported for NIQE and SSEQ.

Metric	Mean	Std	$\hbox {CI}_{{low}}$	$\hbox {CI}_{{high}}$
NIQE	3.12	0.36	2.41	3.83
SSEQ	0.26	0.08	0.10	0.42

**Table 17:** Computational complexity comparison for SOTA methods and proposed methods.

Model	Param. (M)	FLOPs (G)	Memory (MB)
DnCNN	0.56	7.20	520.0
FFDNet	0.49	4.15	320.0
Proposed (W–AE fusion)	0.24	2.85	185.0

Traditional wavelet deinterleavers often struggle with texture preservation, while pure CNN models struggle to cope with the limitations of over-smoothing fine anatomical structures. As confirmed by the ablation study, wavelet decomposition, autoencoder, and CNN components contribute to the performance improvement, contributing to different degrees of improvement.Classical denoising literature indicates that Gaussian noise is often more easily suppressed. However, the proposed method yielded higher SSIM and PSNR values under speckle noise in experimental results. While most models in the current literature focus on a single representation domain (either spatial CNN features or wavelet frequency components), the proposed method can simultaneously utilize complementary features of both domains. The wavelet transform decomposes the image into low and high-frequency components, converting areas dominated by speckle and Gaussian noise into a sparse representation; this allows the CNN to learn noise-free structural patterns more clearly. The autoencoder architecture preserves global context and textural continuity. Optimizing these two representations through a learnable fusion layer has allowed exceeding the performance ceiling (approximately 35 dB PSNR) seen in classical methods. Similar to our approach, more efficiency-oriented results have been observed in models trained with hybrid representations. This is consistent with the signal-dependent nature of speckle noise in medical images; because it contains textural repetitions and structural cues, the proposed patch-based fusion architecture can effectively learn these patterns and better preserve structural information. Consequently, the model converges faster under speckle noise and reconstructs fine anatomical edges with higher accuracy. In contrast, Gaussian noise affects broader spectral components, degrading structural details more aggressively, making denoising relatively difficult. The results show not only quantitative improvement but also significant clinical gains. The fact that speckle noise in ultrasound images carries patterns along with structural information limits the success of classical filtering methods. However, the high SSIM values of the proposed model demonstrate that anatomical folds, tissue boundaries, and echogenicity transitions are preserved more consistently. This feature is a significant advantage in applications where decisions depend on visual accuracy, such as fetal ultrasound. In MRI, it is also important for the more stable preservation of superficial contrast transfer and tumor boundaries, contribution to clearer diagnosis. The features of this study appear to have not yet been adequately addressed in many studies in the literature. When the model’s behavior is examined at different noise levels, it is observed that the proposed architecture converges more quickly and stably, especially against speckle noise. This can be explained by the fact that speckle contains structural repetition, allowing the model to learn these patterns better. Gaussian noise, having a wider spectral distribution, tends to distort structural components more aggressively, which is observed to increase the learning time. This observation reveals that the model does not overfit only one type of noise, but also performs stably under different spectral distortions. Furthermore, a time complexity comparison confirms this finding. Experimentally, denoising under Gaussian noise took longer than under speckle noise, indicating that Gaussian distortion further strains the model learning dynamics (in Table 9 ).

Determining the applicability of the results obtained and the roles they will play in practice is also an important factor in scientific studies.Experimental results and comparative analyses demonstrate that the proposed Wavelet-Enhanced Autoencoder and CNN fusion framework offers a balanced performance between noise reduction achievement, structural detail preservation, and computational cost. Consistently increasing SSIM values under different datasets and noise types reveal that the method provides a strong advantage in preserving clinically significant anatomical structures. This study does not directly focus on task-based clinical assessments, but the statistical stability, structural continuity, and overall robustness findings indicate that the proposed method can be used as a reliable preprocessing step in medical image processing. Therefore, it can particularly support decision-making mechanisms in diagnostic processes.

### Ablation study

When the obtained results are examined from an ablation perspective, the hybrid wavelet and AE approach combines the ability of wavelet-based transforms to effectively suppress noise in high-frequency components with the autoencoder architecture’s ability to learn structural texture and anatomical curves. However, even with some improvements in their individual applications, they are not fully effective. This is because, while wavelet filtering alone can reduce speckle and Gaussian-type noise, it also suppresses some high-frequency details and, in particular, softens fine edge structures, leading to information loss in the image. In contrast, while applying the AE model alone more successfully preserves image structure, it tends to mistakenly learn some components of the noise structure as textural information, potentially causing blurring or artifacts in some regions.

Combination of wavelet filter and autoencoder outputs eliminates the detail loss caused by wavelet filtering alone while also reducing the artifacts that AE alone can cause. Because the learned CNN fusion layer used in the final stage statistically models a nonlinear optimization relationship between wavelet and AE outputs, it can learn data-driven decisions such as which pixels or regions will receive dominant wavelet-based information and which regions will provide more reliable AE estimation. By adaptively combining the strengths of both representations, the proposed method achieves the highest PSNR and SSIM performance, demonstrating successful results in terms of detail preservation, noise suppression, and texture continuity. The ablation study was presented at the noise level for $\sigma$ value is 0.003. Similar trends were observed at other intensities. Ablation study results are shown in Table 18 and Table 19.

**Table 18:** Ablation study for the proposed framework on fetal ultrasound dataset under Gaussian and Speckle noise.

Method	Gaussian noise		Speckle noise	
	PSNR (dB)	SSIM	PSNR (dB)	SSIM
Wavelet only	24.23	0.56	31.42	0.81
Autoencoder only	29.66	0.69	34.85	0.90
Wavelet + AE (avg fusion)	37.46	0.96	39.02	0.96
Proposed (wavelet + AE + CNN)	39.18	0.95	48.12	0.99

**Table 19:** Ablation study for the proposed framework on brain tumor MRI dataset under Gaussian, Speckle and Rician noise.

Method	Gaussian noise		Speckle noise		Rician noise	
	PSNR (dB)	SSIM	PSNR (dB)	SSIM	PSNR (dB)	SSIM
Wavelet only	22.84	0.55	29.18	0.82	15.99	0.79
Autoencoder only	27.11	0.69	32.96	0.90	19.02	0.83
Wavelet + AE (avg fusion)	28.71	0.73	31.22	0.92	20.00	0.85
Proposed (wavelet + AE + CNN)	29.62	0.91	41.21	0.98	29.00	0.90

## Conclusion

In this study, a hybrid method combining a wavelet-based classical filtering approach with a deep learning-based autoencoder and a CNN fusion framework is proposed for the problem of denoising medical images. Experiments on fetal ultrasound and brain tumor images under both Gaussian, speckle and rician noise conditions demonstrate that the proposed method provides significant improvements in PSNR and SSIM metrics compared to the Wiener filter. In particular, the increase in SSIM values demonstrates the method’s success in preserving structural details and supports its potential to improve diagnostic image quality. Experiments have shown that the proposed method provides consistent results even under varying noise levels. In addition to experiments conducted under synthetic noise models, to enhance the reliability of the study, the proposed method was also tested on X-ray images obtained under real clinical conditions and containing natural noise. For this purpose, the GRAZPEDWRI-DX (Wrist Trauma X-ray) dataset, which reflects real shooting conditions and device-dependent noise characteristics, was used. This dataset allows for the analysis of method performance in realistic clinical scenarios without requiring the artificial addition of noise to the images. The results show that the proposed approach exhibits consistent and stable performance not only under controlled synthetic noise conditions but also on real X-ray images. However, it is acknowledged that synthetic noise models cannot fully represent the complex and spatially variable noise statistics of the real world, and therefore, a dataset containing clinical data was used. The results on real clinical data support the robustness and practical applicability of the method. The limitations of the study and the implications for future studies are summarized below:

### Limitations

- Although the study includes three different imaging modalities (ultrasound, MRI and X-ray, the datasets used represent only specific clinical scenarios: fetal head ultrasound, brain tumor MRI and wrist trauma X-ray. Therefore, generalizability to images from different anatomical regions or different imaging protocols has not yet been tested enough.
- Although experiments on real X-ray images were included, part of the evaluation relies on synthetic noise models and full-reference metrics. Therefore, the reported quantitative results should be interpreted as an upper-bound reference, and further validation on large-scale real noisy datasets without ground truth remains an important future direction.
- To control experimental conditions, synthetic noise was added using Gaussian, speckle, and Rician models. However, this synthetic noise may not fully represent the complex and heterogeneous distortions observed in real ultrasound and MRI images, including probe-induced artifacts, low signal-to-noise ratio, and motion-related effects.
- Patch-based training improves performance by providing an advantage in learning local structures, but it introduces additional computational overhead. Furthermore, since the entire image context is divided into patch levels, capturing long-range relationships in modalities with extensive anatomical information, such as MRI, can be difficult.
- The obtained inference times are suitable for the research environment; however, these times need to be reduced in real-time ultrasound or high-speed MRI protocols.

### Future work

- Evaluating the model’s performance in additional imaging fields such as abdominal ultrasound, thyroid ultrasound, musculoskeletal ultrasound, cardiac MRI, and functional MRI will increase its overall applicability.
- Testing on ultrasound recordings containing real speckle or MRI scans with low signal-to-noise ratios will more effectively demonstrate the model’s suitability for clinical conditions.
- Multi-scale attention, canal-spatial attention modules, or deeper residual blocks can enable more efficient integration of wavelet and autoencoder outputs.
- Although the proposed framework consists of multiple stages, each component is computationally lightweight and patch-based, enabling efficient memory usage and parallel execution. Compared to deep end-to-end CNN denoisers, the proposed approach relies on shallower networks and frequency-domain priors, reducing overall model complexity. Nevertheless, real-time clinical deployment depends on hardware configuration and optimized implementations, which will be addressed in future work.
- Techniques such as model compression, quantization, and GPU acceleration can enable real-time use in ultrasound devices or rapid image generation scenarios in MRI.
- Different ultrasound probes or MRI devices have different noise characteristics. Domain adaptation and self-supervised learning approaches can make the method more robust against this diversity.
- In addition, extending the evaluation to fully blind clinical scenarios without reference images and incorporating no-reference quality assessment criteria will further strengthen the applicability of the proposed approach in real-world settings.

## Supplementary Information

Supplementary Information.

## Data Availability

The datasets analyzed in this research is publicly accessible third-party resources hosted on Figshare and Kaggle. It can be retrieved from the following link: https://figshare.com/articles/dataset/ brain_tumor_dataset/1512427 (DOI:10. 6084/m9.figshare.1512427.v5).This dataset is distributed under the terms of the Creative Commons Attribution 4.0 International (CC BY 4.0) license, allowing use, sharing, and reproduction in any format, provided that proper credit is given to the original source. The fetal ultrasound dataset can be accessed from the following link: https://www.kaggle.com/datasets/ankit8467/ fetal-head-ultrasound-dataset-for-image-segment. Third dataset isThe GRAZPEDWRI-DX dataset .It is a publicly available dataset.The dataset is freely accessible under the Creative Commons Attribution 4.0 (CC BY 4.0) licensehttps://www.kaggle.com/datasets/jasonroggy/grazpedwri-dx.The code is avaliable at Zenodo: https://doi.org/10.5281/zenodo.19081947
