# Supplementary material for: Wavelet-enhanced autoencoder based image denoising with CNN fusion
Source: Sci Rep. 2026 Apr 6;16:16503. doi: 10.1038/s41598-026-47179-1 (PMC13216609; doi:10.1038/s41598-026-47179-1)
Supplement: Supplementary file 1 — Supplementary Information. [file 41598_2026_47179_MOESM1_ESM.pdf]

Wavelet-Enhanced Autoencoder Based Image Denoising with CNN Fusion

Iclal Cetin Tas[1*]

[1*]Department of Computer Engineering, Baskent University, 06790, Etimesgut, Ankara, Turkiye.

Corresponding author(s). E-mail(s): icetintas@baskent.edu.tr;

1 Materials-Common medical imaging modalities

Table 1 Common medical imaging modalities, types of noise distribution, and applications(1)

Medical Imaging Modalities	Types of Noise Distribution	Applications
**MRI**	**Thermal Noise, System Noise, Motion Artifacts**	**Brain, spinal cord, joints, soft tissues imaging**
**Ultrasound**	**Speckle Noise, Electronic Noise, Acoustic Noise**	**Obstetrics, cardiology, abdominal imaging**
X-ray	Quantum Noise, Electronic Noise, Scintillation Noise	Fracture detection, chest abnormalities, dental examinations
CT	Electronic Noise, Beam Hardening, Scatter Radiation	Detailed examinations of internal structures, tumor detection
PET	Coincidence Timing Error, Scatter Radiation	Oncology, cardiology, neurology
Mammography	Quantum Noise, Electronic Noise, Motion Artifacts	Breast cancer screening and diagnosis

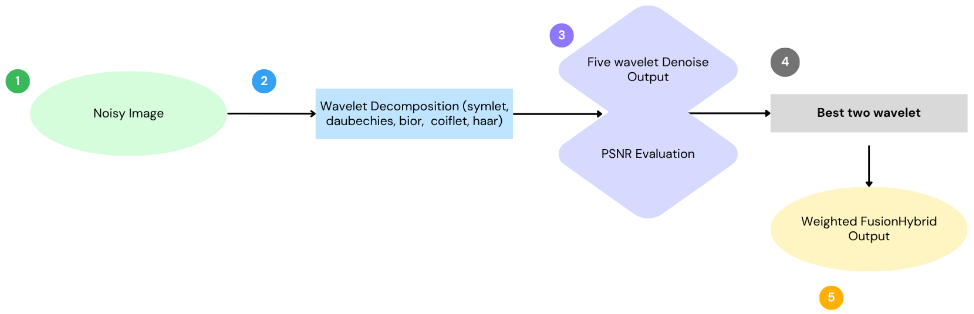

Fig. 1 Flowchart of Hybrid Wavelet Approach

Table 2 Summary of selected wavelet families, key properties, and mathematical formulations

Wavelet	Properties	Mathematical Expression
Sym4	Near-symmetric, orthogonal, reduced phase distortion	$\int t^k \psi(t)\,dt = 0, \quad k = 0, \ldots, N-1$
Db4	Compact support, orthogonal, strong energy compaction	$\int t^k \psi(t)\,dt = 0, \quad k = 0, \ldots, N-1$
Bior3.3	Biorthogonal, symmetric, linear phase, good edge preservation	$\langle \phi_a, \phi_s \rangle = \delta, \quad \langle \psi_a, \psi_s \rangle = \delta$
Coif2	Vanishing moments for both wavelet and scaling functions, high regularity	$\int t^k \psi(t)\,dt = 0, \int t^k \phi(t)\,dt = 0, \quad k = 0, \ldots, N-1$
Haar	Simple, orthogonal, effective for sharp transitions, computationally efficient	$\psi(t) = \begin{cases} 1, & 0 \le t < \frac{1}{2} \\ -1, & \frac{1}{2} \le t < 1 \\ 0, & \text{otherwise} \end{cases}$

2 Wavelet

References

[1] Kumar,R. R. and Priyadarshi,R. , "Denoising and segmentation in medical image analysis: A comprehensive review on machine learning and deep learning approaches," *Multimedia Tools and Applications*, vol. 84, no. 12, pp. 10817–10875, 2024, doi: 10.1007/s11042-024-19313-6.
